# Analysis on the Subdivision of Skilled Mowing Movements on Slopes †

**DOI:** 10.3390/s22041372

**Published:** 2022-02-10

**Authors:** Bo Wu, Yuan Wu, Shoji Nishimura, Qun Jin

**Affiliations:** 1 School of Computer Science, Tokyo University of Technology, 1404-1 Katakuramachi, Hachioji 192-0982, Japan; 2 Advanced Research Center for Human Sciences, Waseda University, 2-579-15 Mikajima, Tokorozawa 359-1192, Japan; kickaha@waseda.jp (S.N.); jin@waseda.jp (Q.J.); 3 Faculty of Human Sciences, Waseda University, 2-579-15 Mikajima, Tokorozawa 359-1192, Japan

**Keywords:** motion analysis, human factors, human information processing, human centric computing

## Abstract

Owing to the aging of the rural population in the hilly and mountainous areas of Japan, mowing on narrow ridges and steep slopes is done manually by the elderly—individuals over 65 years of age. Studies have shown that many accidents that occurred during mowing were caused by workers’ unstable posture, especially when mowing on steep surfaces where there is a high risk of falling. It is necessary to analyze the body movements of mowing workers to elucidate the elements related to the risk of falls. Therefore, in this study, based on a high-precision motion-capture device and a series of experiments with elderly, skilled mowing workers, we focused on the movements of mowing. We sought to identify effective and safe mowing patterns and the factors that lead to the risk of falls. In various mowing styles, compared to the stride (S) and downward (D) mowing patterns, the basic (B) and moving (M) patterns were the most efficient; however, the risk of falls was also the highest among these patterns. While mowing, workers need to pay more attention to their arm strength and take appropriate measures to reduce the risk of falls according to their age and physique. The results can be used as data for the development of fall-detection systems and offer useful insights for the training of new mowing workers.

## 1. Introduction

In the hilly and mountainous areas of Japan, mowing is done using manually operated machines because of the steep gradients of many terraced rice fields [1]. For example, manually driven U-handle-type mowers are the common mowing device used in many slope areas. However, according to a study on agricultural safety [2], mowing is one of the most accident-prone agricultural tasks in Japan. With the aging of the rural population, such mowing works are usually performed by the elderly [3]; hence, it is necessary to consider the safety of elderly workers when mowing on slopes.

According to related surveys [4], approximately 30% of agricultural work accidents were caused by the unstable posture of the worker. Incorrect postures put a heavy burden on a worker’s back, shoulders, and hips, which increases the risk of falls and slips [5]. Studies have shown that falls are associated with reduced postural stability and delayed motor response, especially in the elderly [6]. Therefore, it is important and necessary to analyze their body movements during mowing in detail to determine the characteristics of their movements and fall-prone postures.

With the continuous development of sensors and Internet of Things (IoT) technologies, most human activities, including body movements [7], eye movements [8], and even activities on the Internet [9], can be dynamically acquired and analyzed. Among them, motion-capture devices, which can save a series of human movements as analyzable data through sensors, have been widely used in a variety of studies. Unlike camera-based motion-capture devices [10], wearable motion-capture technology can overcome site constraints and capture the movement of objects in open space in a more detailed manner. By attaching this type of motion-capture device to the elderly people mowing on the slopes, their detailed body-movement data (e.g., angle of joints etc.) can be recorded efficiently for further analysis.

According to relevant studies [11] and related interviews with mowers in our previous works [7,12], personal factors such as age and physique may affect the stability of mowing behaviors, especially for physically demanding tasks like mowing on slopes. To ensure the safety of older workers, it is also necessary to study the effects of personal factors on older workers’ body movements while mowing on slopes.

Studies show that it is very important and necessary to identify the dangerous postures and find effective and safe ways for older people to work on slope mowing. However, the research to date has mainly focused on the analysis of falls in older adults at home, and not much research has been done using motion-capture devices. Therefore, based on a series of experiments by experienced elderly mowing workers using a high-precision motion-capture device (Xsens MVN), this study focuses on the issues of mowing movements on slopes, and aims to identify effective and safe mowing patterns.

Four experienced mowing workers participated in this experiment; they were required to perform normal mowing work. All study participants provided informed consent, and the study design was approved by the ethics review board. The Xsens MVN device and 4 k cameras were used to record the entire mowing actions of the workers for analysis. We also aimed to analyze possible factors influencing the risk of falls while mowing. The results can be accumulated as data for the development of future fall-detection systems and offer useful insights for the training of new mowing workers.

This paper is an extension of the international conference paper [12] we published previously. The remainder of this paper is organized as follows. Issues regarding agricultural accidents analysis, body-movement analysis, and elderly behavior analysis will be presented as an overview of related works in Section 2. In Section 3, the methodology and variable measurements will be described. Based on this, Section 4 provides the datasets, results of related analysis, and discussion; and Section 5 gives the conclusions and future work.

## 2. Related Works

According to the Agricultural Work Safety Information Center of Japan, mowing is one of the most accident-prone agricultural activities in Japan [2]. Accident statistics from the Ministry of Agriculture, Forestry, and Fisheries of Japan indicate that 86.5% of agricultural accidents in 2018 involved workers aged over 65 years [3], and the age of workers is one of the most important factors contributing to agricultural accidents.

Falls in the elderly are associated with reduced postural stability and delayed motor response [6], which means that another important factor influencing the occurrence of accidents is the posture of workers when undertaking agricultural activities. In mowing activities, about 29.5% of accidents were caused by unstable mowing postures [4]. Improper posture places a heavy burden on the worker’s back, shoulders, and hips, which increases the risk of falls and slips.

Consequently, fall prevention in elderly behavior analysis is an active field of study. The survey by Letts et al. on factors affecting the risk of falls in the elderly showed that home hazards such as falls and fires appear to be a significant risk factor in older community-dwelling adults [13].

Several articles analyzed the movements of the elderly, such as the possible relationship between their gaits and falls. For example, through a gait experiment with 597 adults aged 70 years and older, Verghese et al. examined the relationship between speed and six other gait markers and the incidence of falls [14]. To verify whether increased gait variability predicts an increased risk of falls in older adults, Hausdorff et al. measured the stride-to-stride variability of subjects through force-sensitive insoles and collected information on whether they had fallen within a year [15]. The results showed that the stride time variability could predict falls.

There are many comparative studies between older and younger adults. For instance, to examine differences in postural stability and the speed of response between young adults, older adults at low risk of falls, and older adults at high risk of falls, Tucker et al. analyzed their voluntary postural sway movements. They showed that both fall-risk groups had a slower reaction and movement time than the younger group; and that people at high risk of falls had slower reaction and movement times and increased non-target center amplitude during casual swaying [6]. Exploring the incidence of fatal workplace injuries in younger and older workers, Salminen found that younger workers have higher injury rates than older workers, but their workplace fatality rates are lower than those of older workers [16].

Many studies use sensors to collect and analyze data from the elderly. For example, Howcroft et al. developed a fall-risk prediction model for the elderly based on gait data and expected fall occurrence from wearable sensors [17]. Sun et al. assessed the current state of sensing technology in providing objective assessments of fall risk in older adults. They found that four major sensing technologies—inertial sensors, video/depth cameras, pressure sensing platforms, and laser sensing—provide accurate fall risk diagnosis for older adults [18].

Motion-capture devices are one of the important means to analyze human motion, and such devices are used in many fields, such as sports and medical fields. They have been used to analyze the movements of athletes in many studies. Noiumkar et al. studied golf swing in a game using a motion-capture device called mechanical MOCAP system [19]. Cockcroft et al. used a motion-capture device to collect the body-movement data from 10 male competition-level road cyclists for analysis [20]. Unfortunately, the motion data near the pedal and handlebar interface was unacceptable owing to magnetic interference.

Other studies have also applied motion-capture devices to the medical field. For instance, to assess hand rehabilitation, Li et al. provided a simple and intuitive optical motion-capture system, showing a clear and accurate model of hand movements [21]. To provide therapists with detailed and quantifiable data related to patient injury or disability, Kertesz et al. analyzed three-dimensional (3D) representations of patient activity through a motion-capture device, which provided a new method to manage the entire spectrum of the rehabilitation process [22].

In addition to the research that focused on the use of camera-based motion-capture devices, several studies have applied motion-capture devices to human motion analysis in open environments. For example, Burget et al. compared the similarities and differences between subjects performing hand-coordination tasks by asking healthy subjects and patients with Parkinson’s disease to use an acceleration sensor-based motion-capture device [23].

Many studies have focused on the analysis of athletes’ movement. For instance, by having athletes wear a Wi-Fi-based inertial sensing module on their wrist, Abdallah et al. presented a new wearable IoT device capable of monitoring athletes’ movements in real-time [24]. Ivanov et al. presented a wearable electronic system that includes a microcontroller, accelerometers, microsensors, and a heart-rate module for recognizing and controlling the accuracy of athletes’ movements [25].

Studies show that it is very important and necessary to identify the dangerous postures and find effective and safe ways for older people to work on slope mowing. However, the research to date has mainly focused on the analysis of falls in older adults at home, and not much research has been done using motion-capture devices. Therefore, in this study, we focus on the analysis of body movements of skilled mowing workers by motion-capture devices to confirm the safe and effective mowing postures to prevent falls.

## 3. Methodology

### 3.1. Posture Estimation via Joint Angles Calculation

Based on related works and our previous studies [7,12], to identify the elderly workers’ common mowing patterns and possible dangerous postures on slopes in detail, an intuitive way is to calculate and analyze the changing of the angles of their joints while mowing.

In this study, the high-precision motion-capture device Xsens MVN [26] was used to collect the data on elderly workers’ body movements. Xsens MVN is an accelerometer-based wearable motion-capture device that records the 3D coordinates of the joints of the subject every 4 milliseconds (ms). As a full-body motion-capture system, Xsens MVN enables to capture human’s body motion any time and everywhere, in any situation. Xsens MVN’s ultra-small trackers are designed to withstand high impacts, such as rolls and stunts.

In contrast to traditional motion-capture devices, which are based on video camera and 17-joint models, the Xsems MVN device can collect information on 23 joints including spinal segments T12, T8, L5, and L3, and toes as XML files with high precision. The details of collectible joint data are shown in Table 1.

**Figure 1:**
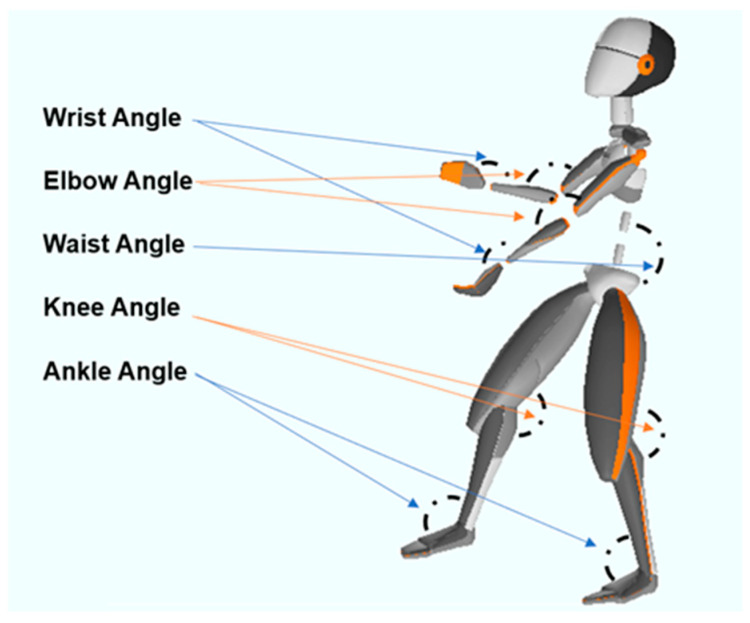
The joints used to represent subjects’ posture.

All joint data collected using the motion-capture device have corresponding 3D coordinates. Based on these, many joint angles can be calculated to represent the posture of the subject. As shown in Figure 1, a total of five types and nine angles are used for analysis, including the wrists, elbows, knees, ankles, and waist.

**Figure 2:**
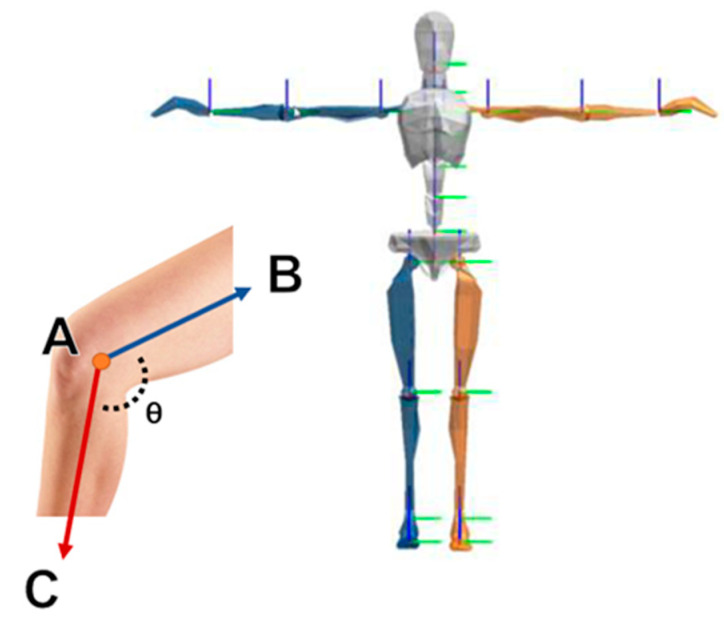
The vectors on ankle joints.

As shown in Figure 2, taking the calculation of the bending angle of the knee as an example, the points *A*, *B*, and *C* represent the joints around the subject’s knee and have coordinates such as *A* (*X_a_, Y_a_, Z_a_*), *B* (*X_b_, Y_b_, Z_b_*), and *C* (*X_c_, Y_c_, Z_c_*). Therefore, from point *A*, two vectors can be calculated as Equations (1) and (2).

(1) $$\;\;\vec{AB}=\left(\;X_{b}-X_{a},\;Y_{b}-Y_{a},\;\;Z_{b}-Z_{a}\right)$$

(2) $$\vec{AC}=\left(\;X_{c}-X_{a},\;Y_{c}-Y_{a},\;\;\;Z_{c}-Z_{a}\;\right)$$

Then, according to these two vectors, the angle of the knee θ can be calculated as Equation (3).

(3) $$Angle\;\theta =\arccos\frac{\vec{AB}\times \vec{AC}\;}{\left|\vec{AB}\right|\times \left|\vec{AC}\right|}\;\;$$

As the motion-capture device Xsens MVN can provide the coordinate metadata of each joint directly, it is only necessary to use the device in actual mowing practice to obtain the required data.

### 3.2. Variables

**Figure 3:**
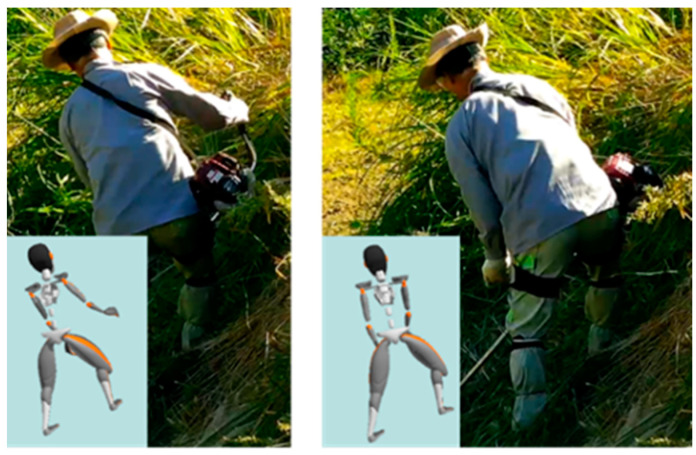
The action of “cutting” on the slope.

As shown in Figure 3, we focus on the analysis of the common action “cutting” on the slope. Therefore, the target analysis range was positioned from the point when the subject lifted the lawn mower to the vertex, to the end of the mowing movement, which means the beginning of the next round of lifting the lawn mower.

Therefore, based on the collected body movements data and the mentioned joint angle calculation method, four variables were calculated for analysis, including the joint angle variation, the standard deviation of waist angle variation, and the moving distance of movement by hands.

As the movement of the user’s wrists, knees, ankles, and other joints is linear in each target analysis range, we calculate the angle variation of each joint as the characteristic quantity.

Because the waist angle variation is not linear in the analysis, the standard deviation was calculated to describe the magnitude of its vibration as the characteristic quantity. The waist is close to the center of gravity of the subject while mowing; hence, the size of the vibration may predict the risk of a fall [27].

As the subjects use their hands to push the mower during the mowing process, the distance of the movement of the hands is usually proportional to the amount of effort they exert; we use the Euclidean formula to calculate the distance of the hands as the characteristic quantity. The distance of the movement of the hands is determined by calculating the coordinate distances of all adjacent frames and adding them, as shown in Equations (4) and (5).

Taking point *i* as an example, the coordinates of the hand before *i* and after *i* are assumed to be *A* (*X_A_*, *Y_A_*, *Z_A_*) and *B* (*X_B_*, *Y_B_*, *Z_B_*), respectively. Therefore, the moving distance $\;D_{i}$ between point *A* to *B* will be:

(4) $$D_{i}=\sqrt{{\left(X_{B}-X_{A}\right)}^{2}+{\left(Y_{B}-Y_{A}\right)}^{2}+{\left(Z_{B}-Z_{A}\right)}^{2}}\;$$

In our data, each frame has a duration of 4 ms; hence, if the target analysis range of one “cutting” action consists of n points, the moving distance of the hand will be:

(5) $$\;\;D=\overset{n-1}{\underset{i=1}{\sum }}D_{i}\;\;\;\;$$

Therefore, as shown in Table 2, a total of 11 variables as the characteristic quantities were considered in this study to model the mowing actions of the subjects on the slope.

## 4. Experiment Results and Discussions

In our previous study [7], we conducted a preliminary study on mowing that analyzed the different mowing patterns, including typical mowing, top-down mowing, and bottom-up mowing. We focus on the typical mowing on slopes, which has the highest risk of the three patterns. This section describes the experimental results, discusses the similarities and differences between the different patterns of typical slope mowing, and further confirms the effect of joint angles variation on the risk of falling. The statistical software IBM Statistical Package for the Social Sciences (SPSS) Statistics 25 was used for related analyses.

### 4.1. Experiments

**Figure 4:**
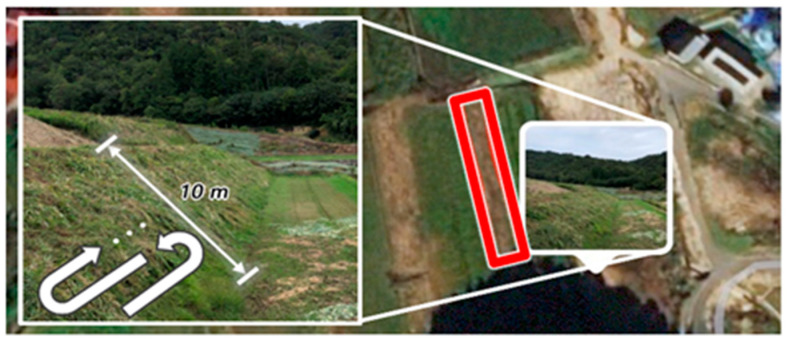
The geography and mowing operation trajectory of the selected experimental area.

Based on the experiments conducted in previous studies [7,12], to obtain the most realistic data on the mowing movements of elderly people, we conducted a new round of experiments on the slope of the paddy field in Kouchi-cho, Higashi-Hiroshima City, Hiroshima Prefecture, Japan. Figure 4 shows the geography and mowing operation trajectory of the selected experimental area.

The area has a large slope angle (slope depth is over 10 m) and the weeds grow so tall that a normal mower robot cannot operate on the area. It requires the mowing worker to make several horizontal trips back and forth to cut the grass manually.

Four experienced mowing workers participated in this experiment. Each participant was over 65 years old and had 10 or more years of slope-mowing experience. The details of the experimental subjects are shown in Table 3.

**Figure 5:**
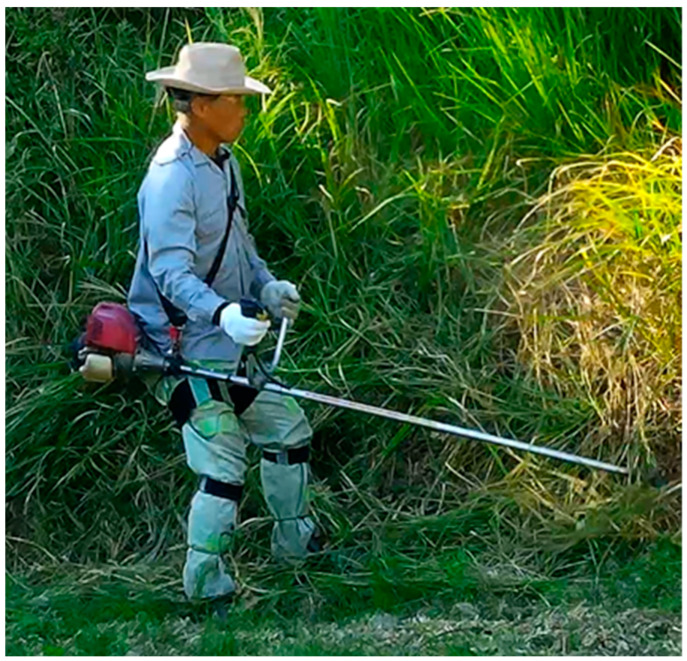
The U-handle-type mower.

All subjects in the experiment used the most common type of mower shown in Figure 5, which is a gasoline-powered U-handle-type mower for a right-hander that allows the operator to turn the front section of the saw teeth using the button on the U-shaped handle of the mower.

All subjects were right-handed and the mowers they used were specifically designed for right-handed people. Moreover, no participants had any cognitive disorder problems, and they were in good health and had normal vision.

To increase the authenticity of the collected data, the subjects were not given any special instructions during the data-collection phase; they were only required to perform the usual mowing work. With confirmation that these additional devices did not interfere with the normal activities of the subjects, all subjects in the trial were equipped with Xsens MVN motion-capture devices. Additional interviews were conducted at the end of all experiments to determine the details could not be obtained visually. In addition to the data collected through the motion-capture device, the movements of the subjects were recorded in real time by the 4k camera on the side and compared to the collected motion data later.

The experiment was completed in September 2020, and a supplementary experiment was carried out in September 2021. A total of 627 sets of mowing movements on slopes were collected for analysis. To make the 3D model more accurate, we also collected additional physical and personal data of the subjects as shown in Table 4.

### 4.2. Analysis for Different Patterns of Mowing on Slopes

**Figure 6:**
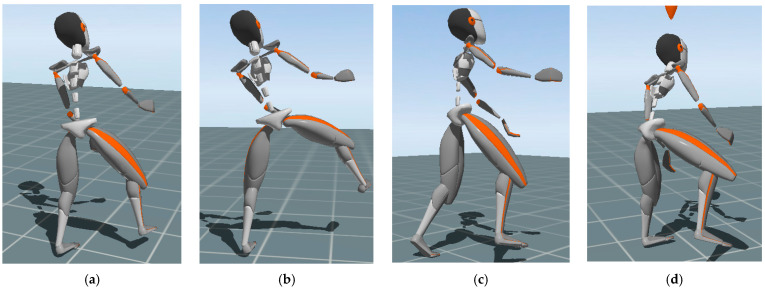
Different mowing patterns of “typical mowing” on the slope. (**a**) Basic mowing. (**b**) Stride mowing. (**c**) Moving mowing. (**d**) Downward mowing.

Through experimental correlation image (especially the lower-body posture of workers) analysis and interviews with subjects, we found that the classic mowing action on slopes can be divided into four different patterns. As shown in Figure 6, we classified the movements of the subjects into the following four patterns based on their basic posture: basic, stride, moving mowing, and downward mowing actions.

Basic mowing action refers to the mowing action performed on the weeds in front of the subject while standing naturally with both feet. Stride mowing action refers to the mowing action where the subject takes a large stride with one foot in order to maintain stability.

Moving mowing motion refers to the mowing action when the subject moves his or her position to perform the mowing task better. Stride and moving motions are both performed while mowing weeds in front of the subject. Finally, downward mowing motion refers to the mowing motion performed to the weeds below the subject, with the subject’s feet in their natural state. As per the mowing operation trajectory shown in Figure 4, the subjects did not mow the weeds above them, to avoid danger.

Through basic statistics, as shown in Table 5, we found that among all the data, various actions remained on the order of 50–100, except for the “basic mowing action” (B) action, which was the most common. Therefore, we focus on the similarities and differences between various other patterns and the B action.

Based on the chi-square analysis of the data, we learned that target variables do not conform to the normal distribution. Therefore, to distinguish the similarities and differences between these patterns, the Kruskal–Wallis one-way ANOVA is used to analyze the difference between the characteristic quantities of these types of actions.

As shown in Table 6, the results of the Kruskal–Wallis one-way ANOVA show that significant differences are confirmed in all the measures we considered. The results prove that our classification for mowing actions is correct, and that each of the four different patterns has its own different characteristics. However, as there are four patterns involved in the analysis, we will focus on the results of the post hoc comparison.

We will discuss the similarities and differences of each of the post hoc results in three parts, including the upper body, lower body, and the waist vibration and mowing distance of hands.

#### 4.2.1. Post Hoc Results for Upper-Body Mowing Actions

**Figure 7:**
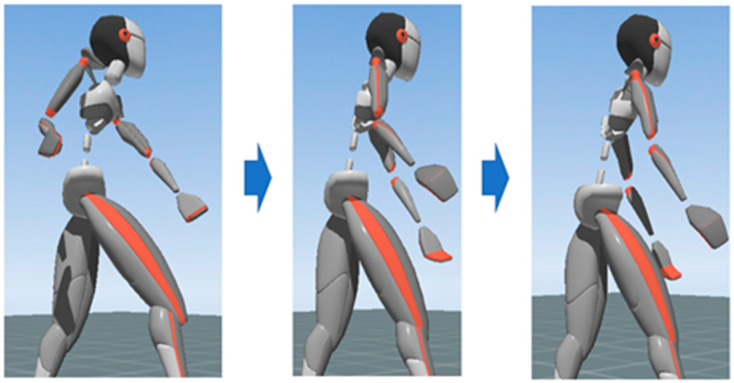
The upper-body mowing action.

Through post-event questionnaires and interviews, we learned that in slope mowing, the body joint movements determine how the worker exerts his or her force and technique. As the way of mowing involves lifting the mower and exerting the force diagonally downward from right to left, the movement of the worker’s left and right hands and feet are different. As shown in Figure 7, in terms of the variation of joint angles, the worker’s joint angles (e.g., elbow and knee) undergo a process from flexion to flatness when mowing. It can usually be assumed that the larger this amount of variation is, the more energy the worker outputs.

Therefore, for the upper-body joints, the results of post hoc analysis show that although there is significant difference in almost all movements between the four patterns, similarity also appears in some measures.

**Figure 8:**
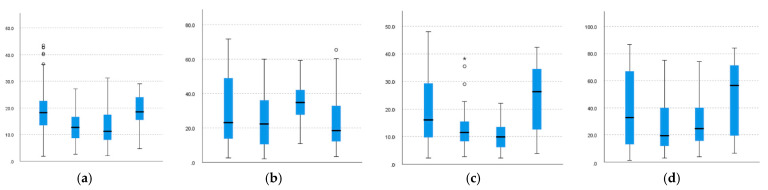
Comparison of upper-body joint angle variation. (**a**) VRelbow. (**b**) VRwrist. (**c**) VLelbow. (**d**) VLwrist.

As shown in Figure 8 and Table 7, for the angle variation of the right elbow, similarities are observed between the patterns of the B–M and S–D pairs; the same results are also observed for the angle variation of the left elbow. The results also indicate that the B–M pair has a higher level than the S-D pair in the angle variation. A possible explanation for this result is that, in contrast to the S and D patterns, which need to change the upper posture to mow, the difference between B and M lies mainly in moving or not, the workers have similar upper-body movements and can output a greater force.

The right and left wrists need to be discussed separately. Through our investigation, the right hand is mainly responsible for pushing the mower to move, whereas the left hand is more responsible for lifting the mower.

For the right wrist, similarities are observed between the patterns of the B–M, B–S, and S–M pairs. Pattern S has a higher level than others in the angle variation. One possible explanation is that the right hand of the worker is primarily responsible for pushing the mower during mowing, especially when mowing downward, in which it requires more force to push the mower because the workers need to change the way they hold the control lever.

For the left wrist, similarities are observed between the patterns of B–D and S–D. Pattern M has a higher level than others in the angle variation. A possible explanation is that the worker needs to exert more force on the left hand to lift the mower when moving with pattern M.

#### 4.2.2. Post Hoc Results for Lower-Body Mowing Actions

**Figure 9:**
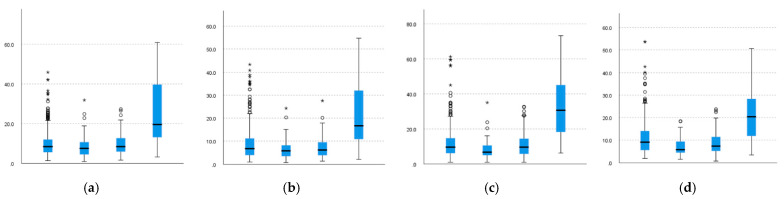
Comparison of lower-body joint angle variation. (**a**) VRknee. (**b**) VRankle. (**c**) VLknee. (**d**) VLankle.

As shown in Figure 9 and Table 8, the results of post hoc analysis show that similarities are also observed between lower-body actions. Unlike the upper body, the lower-body movements become more undulating due to susceptibility to topography. Outliers as an element to reflect environmental impact are not removed from the analysis in this study because they are real data.

It is important to note that because of the characteristics of the pattern M, which is different from the other three patterns, only the data of M (having higher values than others) are listed and not compared and discussed in this paper. The comparison results of the other three patterns are as follows.

For the right knee and ankle, similarities are observed between the patterns of the B–S, B–D, and S–D pairs. As the standard pattern for mowing on a slope is to stand firmly with the right foot in front and the left foot at the back, the results mean that in all patterns, the right foot may be primarily responsible for maintaining balance and not for exerting force for mowing.

For the left knee, the similarity is observed between the pattern B–D, and for the left ankle, the similarity is observed between the patterns B–D and S–D, that each of the values of the S pattern is smaller than the others. The possible explanation is that the left foot, as the weight-bearing foot, does not change much in terms of force under pattern B and D, but in pattern S, owing to the forward shift of the center of gravity, its weight-bearing force is partially reduced.

#### 4.2.3. Post Hoc Results for Waist Vibration and Mowing Distance of Hands

**Figure 10:**
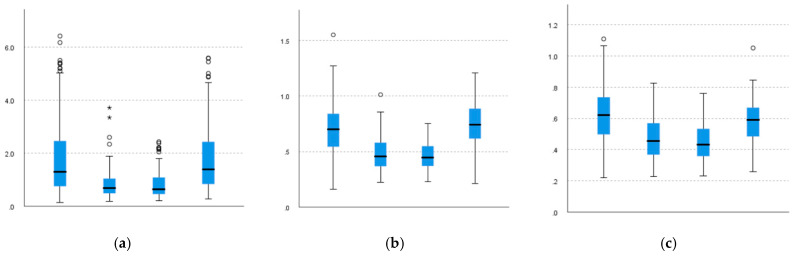
Comparison of waist vibration and moving distance of hands. (**a**) STDwaist. (**b**) DRight. (**c**) DLeft.

Lastly, for the post hoc results for waist vibration and moving distance of hands, as shown in Figure 10 and Table 9, similarity is also observed. In general, greater waist vibration represents a higher risk of falling, whereas an increase in the moving distance of hands can laterally indicate an increase in the force used.

For waist vibration and the moving distance of right hand and left hand, the analysis results show that similarities are observed between the patterns of the B-M and S-D pairs, which are similar to the results of the upper-body elbow analysis. If the explanation regarding the elbows’ angle variation (workers output more power in B and M patterns) is correct, this means that the output of greater force is subject to a greater risk of falling. The results of the moving distance by both hands corroborate this hypothesis.

Based on the analysis for different patterns of mowing on slopes, the results of the Kruskal–Wallis one-way ANOVA and related post hoc analysis show that among the four patterns, the B pattern has the highest number and, like the M pattern, allows workers to mow with maximum effort. Workers in the S and D patterns have to use slightly less force to mow to maintain balance. Therefore, if the conditions allow, the B and M patterns are recommended to use to increase efficiency when mowing on slopes. However, the results also show that when using the B and M patterns for mowing, workers need to be aware of falls, as higher efficiency means higher risk.

### 4.3. Analysis of Falling Risk of Elderly Workers When Mowing on Slopes

From the conclusion of Section 4.1, posture is an important factor that affects the use of force during mowing. Therefore, based on pattern B with the largest amount of data, we attempted to analyze the factors affecting the safety of mowing on slopes via the method of stepwise regression. In addition to the variation of the angle of joints, individual factors of workers (e.g., age, experience, arm span etc.) were also considered as important influences.

Thus, in the stepwise regression, the STDwaist, which represents the vibration of the waist joint, was set as the dependent variable, and the other measures were set as the independent variables.

**Figure 11:**
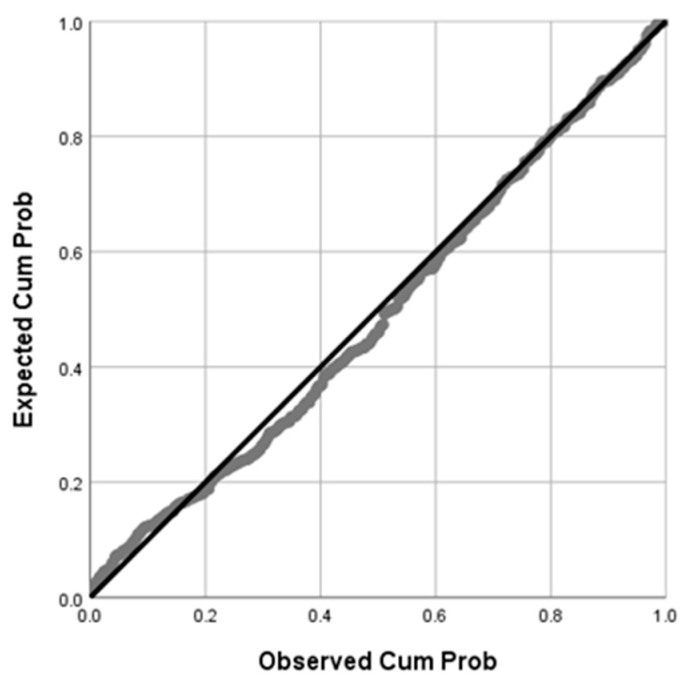
Normal P–P plot of regression standardized residual for STDwaist.

To meet the conditions of stepwise regression analysis, after correlation pretreatment, as shown in Figure 11, we determined that the dependent variable STDwaist in the analysis conforms to normal distribution.

As shown in Table 10, the results of the stepwise regression model show that a total of five variables (VLelbow, VRelbow, VLwrist, Arm span and Age) significantly affect the dependent variables STDwaist, with the values of R: 0.847 and R2: 0.714 (*p*-value < 0.01).

In terms of the magnitude of influence with the direction positive, the biggest two are the angle variation of the left elbow and right elbow, which mean that the force exerted while mowing is mainly by the angle variation of the joint of the worker’s elbow; the greater the angle variation, the greater the power output. However, sometimes it also means a higher waist vibration, which may cause a fall while mowing on the slope. Positive effects were also observed on the left wrist that may suggest that the force of holding the mower in the left hand also affects the vibration in the waist and further affects the possibility of falling. The results are consistent with those discussed in Section 4.1.

The results show that in addition to the left and right elbow and left wrist, arm span and age also play a significant role as influencing factors. The effects are bigger, but the direction of the effects is negative.

The results of arm span analysis mean that the longer a person’s arm span, the less vibration their waist has while mowing. As the length of a person’s arms is often proportional to his physical size, this means that a worker who is not sufficiently strong may have to exert more effort to mow the grass, and may face a higher risk of falling while mowing on a slope.

Contrary to expectations, the results on the effect of workers’ age showed that elder workers had more stable waists while mowing on slopes. Because the experimental subjects had similar mowing experiences, one possible explanation is that older workers were more concerned with their body balance during work. This means that without physical limitations, workers over the age of 65 may have a safer body motion for slope mowing.

Based on the analysis of falling risk of elderly workers when mowing on slopes, the results of stepwise regression analysis are as follows. To reduce the possibility of falling while mowing, workers need to pay attention to the use of arm strength and take appropriate measures to reduce the risk of falls according to their age and physique.

## 5. Conclusions

To identify the most effective and safe mowing patterns and the possible influence factors of fall risk while mowing on slopes, in this study a series of experiments were conducted to collect data and analyze the body movements of workers during mowing.

Based on 627 sets of motion data collected from four elderly workers, the Kruskal–Wallis one-way ANOVA and the related post hoc analysis show significant differences in all the measures among the mowing patterns B, S, D, and M. At the same time, the patterns B and M have higher efficiency when mowing on slopes, but also carry a higher risk of falling.

The results of stepwise regression indicate that a relatively old worker with a healthy physique may have a safer mowing motion on the slope. To reduce the risk of falling, the workers need to pay attention to their arms when cutting grass on a slope. The results can be used as data for the development of future fall-detection systems and offer useful insights for the training of new mowing workers.

Due to the risk of mowing on steep slopes, more subjects over the age of 65 could not be invited to join the related experiments. The discussion in this paper assumes that workers with more than 20 years of mowing experience will have safer and more efficient postures than novices.

In future work, we plan to break down the mowing action in terms of frequency, consider more human-related data such as mowing workers’ eye movements and invite more workers with different characteristics (e.g., gender) to conduct experiments. We are also considering the use of deep-learning technology to predict the workers’ risk of falling.

## Figures and Tables

**Table 1:** The details of collectable joints by Xsens MVN.

No.	Label	Detail
1	Pelvis	Pelvis
2	L5	L5
3	L3	L3
4	T12	T12
5	T8	T8
6	Neck	Neck
7	Head	Head
8	Rshoulder	Right shoulder
9	RUArm	Right upper arm
10	Rforearm	Right forearm
11	Rhand	Right hand
12	Lshoulder	Left shoulder
13	LUArm	Left upper arm
14	Lforearm	Left forearm
15	LHand	Left hand
16	RULeg	Right upper leg
17	RLLeg	Right lower leg
18	Rfoot	Right foot
19	RToe	Right toe
20	LRLeg	Left upper leg
21	LLLeg	Left lower leg
22	Lfoot	Left foot
23	Ltoe	Left toe

**Table 2:** Details of measures for analysis.

No.	Measures	Detail
1	DRight	Moving distance of right hand
2	DLeft	Moving distance of left hand
3	VRelbow	Variation of right elbow joint angle
4	VRwrist	Variation of right wrist joint angle
5	VLelbow	Variation of left elbow joint angle
6	VLwrist	Variation of left wrist joint angle
7	VRknee	Variation of right knee joint angle
8	VRankle	Variation of right ankle joint angle
9	VLknee	Variation of left knee joint angle
10	VLankle	Variation of left ankle joint angle
11	STDwaist	Vibration of lumbar joint (standard variance of lumbar angle changing)

**Table 3:** Details of subjects in the experiment.

No.	Gender	Age	Experience	Arm Span
1	Male	74	Around 40 years	163
2	Male	68	Around 40 years	170
3	Male	66	Around 40 years	161
4	Male	68	Around 8 years	167

**Table 4:** Details of physical and personal measures for analysis.

No.	Type	Data
1	Physical data	Body height
		Foot or shoe length
		Shoulder height
		Shoulder width
		Arm span
		Hip height
		Hip width
		Knee height
		Ankle height, etc.
2	Personal data	Gender
		Age
		Mowing experience
		Medical history
		Work evaluation, etc.

**Table 5:** Details of measures for analysis.

No.	Pattern	Abb.	Number
1	Basic mowing action	B	406
2	Stride mowing action	S	59
3	Downward mowing action	D	93
4	Moving mowing action	M	69

**Table 6:** Results of Kruskal–Wallis one-way ANOVA.

Measures	Pattern	N	Mean Rank	Chi-Square	df	Sig. (2-Tailed)
VRelbow	B	406	346.07	79.37	3	0.00
	S	59	207.41			
	D	93	197.19			
	M	69	373.90			
VRwrist	B	406	314.82	27.75	3	0.00
	S	59	261.48			
	D	93	387.70			
	M	69	254.74			
VLelbow	B	406	340.32	78.76	3	0.00
	S	59	235.34			
	D	93	188.13			
	M	69	396.06			
VLwrist	B	406	321.46	26.80	3	0.00
	S	59	243.09			
	D	93	270.86			
	M	69	388.90			
VRknee	B	406	296.26	81.41	3	0.00
	S	59	248.78			
	D	93	298.28			
	M	69	495.36			
VRankle	B	406	300.45	93.55	3	0.00
	S	59	248.32			
	D	93	271.55			
	M	69	507.10			
VLknee	B	406	296.16	115.42	3	0.00
	S	59	211.41			
	D	93	301.68			
	M	69	523.30			
VLankle	B	406	309.91	82.58	3	0.00
	S	59	222.22			
	D	93	264.47			
	M	69	483.30			
STDwaist	B	406	346.11	71.86	3	0.00
	S	59	210.97			
	D	93	202.90			
	M	69	362.88			
DRight	B	406	357.21	140.16	3	0.00
	S	59	176.37			
	D	93	156.02			
	M	69	390.36			
DLeft	B	406	359.74	101.38	3	0.00
	S	59	203.15			
	D	93	177.02			
	M	69	324.25			

**Table 7:** Post hoc testing of upper-body joints.

Title 1	Sample 1 –Sample 2	Test Statistic	Std. Error	Std. Test Statistic	Sig.	Adj. Sig.
VRelbow	D–S	10.21	30.15	0.34	0.74	1.00 ^n.s.^
	D–B	148.87	20.82	7.15	0.00	0.00
	D–M	−176.71	28.78	−6.14	0.00	0.00
	S–B	138.66	25.24	5.49	0.00	0.00
	S–M	−166.49	32.12	−5.18	0.00	0.00
	B–M	−27.83	23.59	−1.18	0.24	1.00 ^n.s.^
VRwrist	M–S	6.74	32.12	0.21	0.83	1.00 ^n.s.^
	M–B	60.08	23.59	2.55	0.01	0.07 ^n.s.^
	M–D	132.96	28.78	4.62	0.00	0.00
	S–B	53.35	25.24	2.11	0.04	0.20 ^n.s.^
	S–D	−126.22	30.15	−4.19	0.00	0.00
	B–D	−72.88	20.82	−3.50	0.00	0.00
VLelbow	D–S	47.21	30.15	1.57	0.12	0.70 ^n.s.^
	D–B	152.19	20.82	7.31	0.00	0.00
	D–M	−207.93	28.78	−7.22	0.00	0.00
	S–B	104.98	25.24	4.16	0.00	0.00
	S–M	−160.72	32.12	−5.00	0.00	0.00
	B–M	−55.74	23.59	−2.36	0.02	0.11 ^n.s.^
VLwrist	S–D	−27.78	30.15	−0.92	0.36	1.00 ^n.s.^
	S–B	78.37	25.24	3.11	0.00	0.01
	S–M	−145.81	32.12	−4.54	0.00	0.00
	D–B	50.60	20.82	2.43	0.02	0.09 ^n.s.^
	D–M	−118.04	28.78	−4.10	0.00	0.00
	B–M	−67.44	23.59	−2.86	0.00	0.03

^n.s.^ Not significant.

**Table 8:** Post hoc testing of lower-body joints.

Title 1	Sample 1 –Sample 2	Test Statistic	Std. Error	Std. Test Statistic	Sig.	Adj. Sig.
VRknee	S–B	47.48	25.24	1.88	0.06	0.36 ^n.s.^
	S–D	−49.50	30.15	−1.64	0.10	0.60 ^n.s.^
	S–M	−246.58	32.12	−7.68	0.00	0.00
	B–D	−2.02	20.82	−0.10	0.92	1.00 ^n.s.^
	B–M	−199.11	23.59	−8.44	0.00	0.00
	D–M	−197.08	28.78	−6.85	0.00	0.00
VRankle	S–D	−23.23	30.15	−0.77	0.44	1.00 ^n.s.^
	S–B	52.13	25.24	2.07	0.04	0.23 ^n.s.^
	S–M	−258.78	32.12	−8.06	0.00	0.00
	D–B	28.90	20.82	1.39	0.17	0.99 ^n.s.^
	D–M	−235.55	28.78	−8.18	0.00	0.00
	B–M	−206.65	23.59	−8.76	0.00	0.00
VLknee	S–B	84.75	25.24	3.36	0.00	0.01
	S–D	−90.27	30.15	−2.99	0.00	0.02
	S–M	−311.90	32.12	−9.71	0.00	0.00
	B–D	−5.52	20.82	−0.27	0.79	1.00 ^n.s.^
	B–M	−227.14	23.59	−9.63	0.00	0.00
	D–M	−221.63	28.78	−7.70	0.00	0.00
VLankle	S–D	−42.25	30.15	−1.40	0.16	0.97 ^n.s.^
	S–B	87.69	25.24	3.47	0.00	0.00
	S–M	−261.08	32.12	−8.13	0.00	0.00
	D–B	45.44	20.82	2.18	0.03	0.18 ^n.s.^
	D–M	−218.83	28.78	−7.60	0.00	0.00
	B–M	−173.40	23.59	−7.35	0.00	0.00

^n.s.^ Not significant.

**Table 9:** Post hoc testing of waist vibration and moving distance of hands.

Title 1	Sample 1 –Sample 2	Test Statistic	Std. Error	Std. Test Statistic	Sig.	Adj. Sig.
STDwaist	D–S	8.06	30.15	0.27	0.79	1.00 ^n.s.^
	D–B	143.21	20.82	6.88	0.00	0.00
	D–M	−159.98	28.78	−5.56	0.00	0.00
	S–B	135.15	25.24	5.36	0.00	0.00
	S–M	−151.92	32.12	−4.73	0.00	0.00
	B–M	−16.77	23.59	−0.71	0.48	1.00 ^n.s.^
DRight	D–S	20.35	30.05	0.68	0.50	1.00 ^n.s.^
	D–B	199.86	20.77	9.62	0.00	0.00
	D–M	−234.34	28.69	−8.17	0.00	0.00
	S–B	179.50	25.17	7.13	0.00	0.00
	S–M	−213.99	32.02	−6.68	0.00	0.00
	B–M	−34.49	23.52	−1.47	0.14	0.86 ^n.s.^
DLeft	D–S	26.13	30.05	0.87	0.39	1.00 ^n.s.^
	D–M	−147.23	28.69	−5.13	0.00	0.00
	D–B	181.40	20.77	8.74	0.00	0.00
	S–M	−121.09	32.02	−3.78	0.00	0.00
	S–B	155.27	25.17	6.17	0.00	0.00
	M–B	34.18	23.52	1.45	0.15	0.88 ^n.s.^

^n.s.^ Not significant.

**Table 10:** Results of stepwise regression of STDwaist.

Model ^note^	Unstandardized Coefficients		Standardized Coefficients	t	Sig.
	B	Std. Error	Beta		
(Constant)	32.79	1.69		19.4	0.00
VLelbow	0.02	0.01	0.22	3.64	0.00
ArmSpan	−0.15	0.01	−0.41	−14.01	0.00
VLwrist	0.02	0.00	0.43	7.69	0.00
Age	−0.12	0.02	−0.24	−7.19	0.00
VRelbow	0.02	0.01	0.11	3.57	0.00

^note^ R = 0.847, R2 = 0.714.

## Data Availability

Not applicable.
